# End-of-treatment anti-HBs levels and HBeAg status identify durability of HBsAg loss after PEG-IFN discontinuation

**DOI:** 10.3389/fcimb.2023.1120300

**Published:** 2023-02-24

**Authors:** Yifei Guo, Jiajia Han, Yongmei Zhang, Chengmeng Jin, Yao Zhang, Jingjing He, Shiqi Chen, Yue Guo, Yanxue Lin, Fahong Li, Feifei Yang, Zhongliang Shen, Richeng Mao, Haoxiang Zhu, Jiming Zhang

**Affiliations:** ^1^ Department of Infectious Diseases, Shanghai Key Laboratory of Infectious Diseases and Biosafety Emergency Response, Shanghai Institute of Infectious Diseases and Biosecurity, National Medical Center for Infectious Diseases, Huashan Hospital, Fudan University, Shanghai, China; ^2^ Department of Medical Microbiology and Parasitology, School of Medical Sciences, Fudan University, Shanghai, China; ^3^ Key Laboratory of Medical Molecular Virology (MOE/NHC/CAMS), Shanghai Frontiers Science Center of Pathogenic Microorganisms and Infection, School of Basic Medical Sciences, Shanghai Medical College, Fudan University, Shanghai, China; ^4^ Department of Infectious Diseases, Jing’An Branch of Huashan Hospital, Fudan University, Shanghai, China

**Keywords:** chronic hepatitis B, HBsAg reversion, functional cure, PEG-IFN, HBeAg, anti-HBs

## Abstract

**Background:**

Hepatitis B surface antigen (HBsAg) loss, namely, the functional cure, can be achieved through the pegylated interferon (PEG-IFN)-based therapy. However, it is an unignorable fact that a small proportion of patients who achieved functional cure develop HBsAg reversion (HRV) and the related factors are not well described.

**Methods:**

A total of 112 patients who achieved PEG-IFN-induced HBsAg loss were recruited. HBV biomarkers and biochemical parameters were examined dynamically. HBV RNA levels were assessed in the cross-sectional analysis. The primary endpoint was HRV, defined as the reappearance of HBsAg after PEG-IFN discontinuation.

**Results:**

HRV occurred in 17 patients during the follow-up period. Univariable analysis indicated that hepatitis B e antigen (HBeAg) status, different levels of hepatitis B surface antibody (anti-HBs), and hepatitis B core antibody (anti-HBc) at the end of PEG-IFN treatment (EOT) were significantly associated with the incidence of HRV through using the log-rank test. Additionally, time-dependent receiver operating characteristic (ROC) analysis showed that the anti-HBs was superior to anti-HBc in predictive power for the incidence of HRV during the follow-up period. Multivariable Cox proportional hazard analysis found that anti-HBs ≥1.3 log_10_IU/L (hazard ratio (HR), 0.148; 95% confidence interval (CI), 0.044-0.502) and HBeAg negativity (HR, 0.183; 95% CI, 0.052-0.639) at EOT were independently associated with lower incidence of HRV. Cross-sectional analysis indicated that the HBV RNA levels were significantly correlated with the HBsAg levels in patients with HRV (r=0.86, p=0.003).

**Conclusions:**

EOT HBeAg negativity and anti-HBs ≥1.3 log_10_IU/L identify the low risk of HRV after PEG-IFN discontinuation.

## 1 Introduction

Chronic hepatitis B virus (HBV) infection remains a major global health issue, affecting approximately 250 million people (WHO., 2017). Currently, hepatitis B surface antigen (HBsAg) loss with or without hepatitis B surface antibody (anti-HBs) is considered as the optimal endpoint for antiviral treatment and referred to as ‘functional cure’, but it is rarely achieved, putting patients at risk for severe liver diseases, including cirrhosis, hepatic decompensation, and hepatocellular carcinoma (HCC) (WHO, 2017).

Nucleos(t)ide analogues (NAs) are the first-line treatment and can provide sustained suppression of HBV replication (Sarin et al., 2016; Terrault et al., 2016; European Association for the Study of the Liver, 2017), but previous studies suggested that patients with pegylated interferon (PEG-IFN)-based therapy exhibited higher rates of achieving HBsAg loss compared to patients with NAs therapy (Marcellin et al., 2016; Yip et al., 2017; Wu et al., 2020; Tout et al., 2021). Unfortunately, a small proportion of patients who achieved HBsAg loss develop HBsAg reversion (HRV) during follow-up. The cumulative probability of HRV was 9.66% for 597 weeks in patients treated with interferon (IFN) (Wu et al., 2020). To date, however, fewer studies have focused on predicting HRV in patients with PEG-IFN-induced HBsAg loss.

Considering the likelihood of HRV and subsequent adverse outcomes, in this study, we focused on evaluating the factors affecting HRV after PEG-IFN discontinuation. The results of our study could be useful for clinicians to select the appropriate time to discontinue PEG-IFN treatment for patients who achieved HBsAg loss.

## 2 Materials and methods

### 2.1 Patients

**Figure 1:**
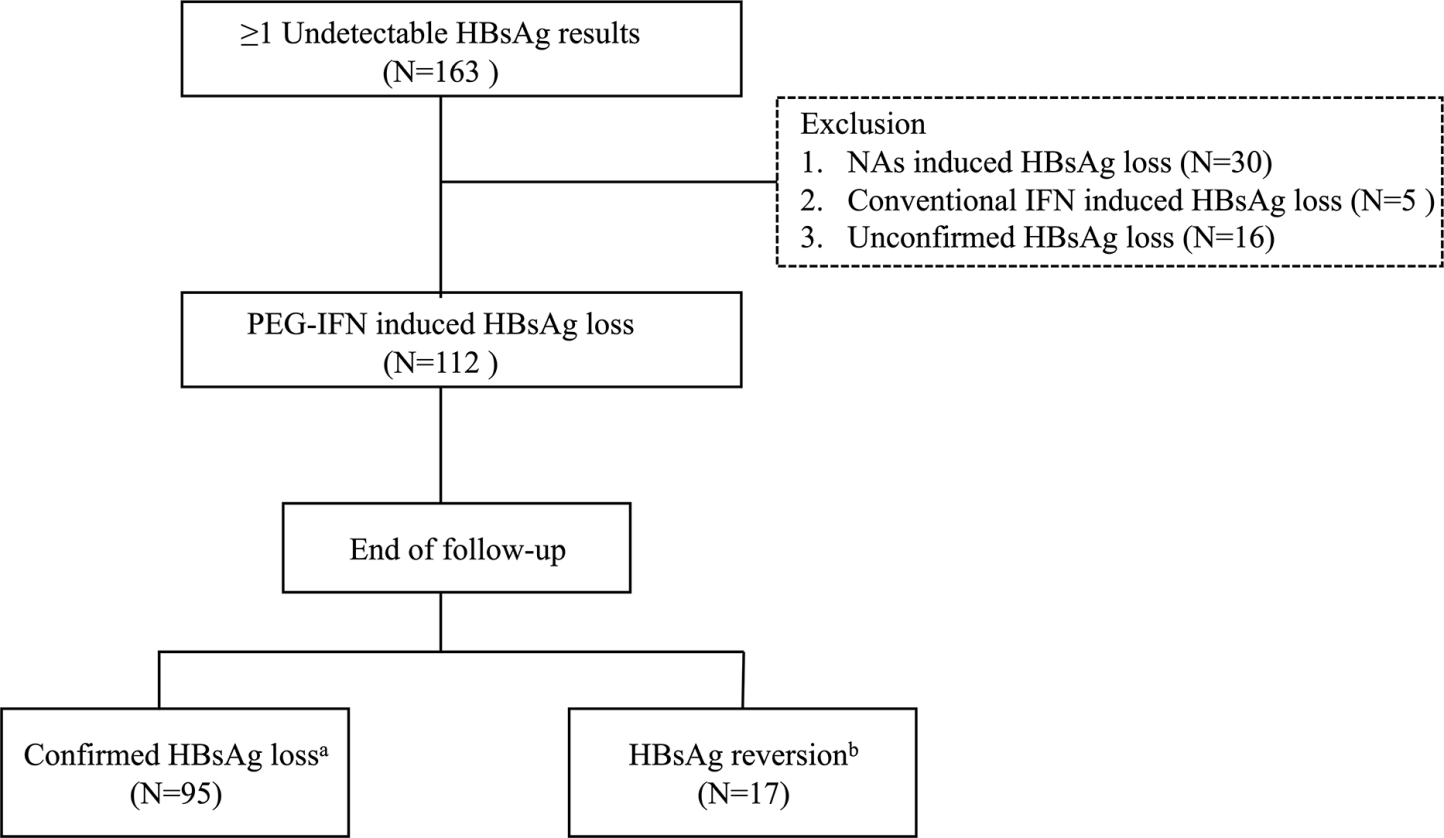
Flowchart of patients’ selection process. HBsAg, hepatitis B surface antigen; NA, nucleos(t)ide analogue; IFN, interferon; PEG-IFN, pegylated interferon. ^a^Two negative HBsAg results (<0.05 IU/mL) at least 6 months apart. ^b^Reappearance of HBsAg after HBsAg loss.

A retrospective cohort study was performed in Huashan Hospital from Jan 2014 to Dec 2019. A total of 163 chronic hepatitis B (CHB) patients who had at least one undetectable HBsAg result were consecutively enrolled, and those 112 patients who achieved PEG-IFN-induced HBsAg loss were ultimately analyzed. Exclusion criteria were NAs-induced HBsAg loss; conventional IFN-induced HBsAg loss; unconfirmed HBsAg loss (**Figure 1**). The study was approved by the Ethics Committee of Huashan Hospital of Fudan University and carried out in accordance with the current version of the Helsinki Declaration.

### 2.2 Laboratory measurements

Liver biochemical parameters were determined by a biochemistry analyzer (7600 Series; Hitachi, Tokyo, Japan). Platelet was measured by Sysmex XN-2000 (Kobe, Japan). Serum HBsAg, anti-HBs, hepatitis B e antigen (HBeAg), hepatitis B e antibody (anti-HBe), and hepatitis B core antibody (anti-HBc) were detected using an enzyme-linked immunosorbent assay kit (ARCHITECT i2000 SR; Abbott Architect, USA). Serum HBsAg were retested (Roche Cobas e602; Roche, Switzerland) when it exceeded the upper linearity limit (250 IU/mL). HBV DNA was quantified by using a real-time PCR assay (DAAN Diagnostics, Guangzhou, China). Detection limits of HBsAg, anti-HBs, HBeAg, anti-HBe, anti-HBc, and HBV DNA were 0.05 IU/mL, 10 IU/L, 1 S/CO, 1 S/CO, 1 S/CO, 500 IU/mL, respectively. The upper limit of normal (ULN) of ALT has been defined as 40 U/L for women and 50 U/L for men. The ULN of bilirubin has been defined as 21 μmol/L for women and 26 μmol/L for men. The normal range of albumin was 40-55 g/L. In addition, hepatitis B core-related antigen (HBcrAg) was not detected due to the lack of serum samples.

### 2.3 HBV RNA concentration measurement

HBV RNA, as reverse transcribed pre-genomic HBV RNA, was extracted from 200 μL serum with the nucleic acid extraction or purification kit (magnetic beads method) (Sansure Biotech, Changsha, China). Next, the extraction was treated with DNase I (Thermo Fisher Scientific, Waltham, MA, USA). Finally, DNase-I-treated HBV RNA was quantitatively measured with the HBV RNA quantitative kit (Sansure Biotech, no.010106025). The detection limit of the assay was 100 copies/mL. HBV RNA detection was conducted in 64 patients, not all patients, due to the lack of serum samples.

### 2.4 Definitions and endpoints

The primary outcome was the development of HRV. Follow-up duration was measured from the date of the end of PEG-IFN treatment (EOT) to the date of HRV or the last follow-up visit. Consolidation treatment duration was measured from the date of HBsAg loss to the date of EOT. Confirmed HBsAg loss (CHL) was defined as two negative HBsAg results (<0.05 IU/mL) at least 6 months apart; HRV was defined as the reappearance of HBsAg after HBsAg loss. PEG-IFN monotherapy was defined as PEG-IFN therapy in naïve chronic hepatitis B (CHB) patients. Add-on PEG-IFN was defined as combination therapy after at least 48 weeks of nucleot(s)ide therapy. Switch-to PEG-IFN was defined as PEG-IFN monotherapy in patients who received NAs for at least 48 weeks.

### 2.5 Statistical analysis

Continuous variables were expressed as mean (interquartile range [IQR]). Categorical variables were expressed as counting and percentage. Comparison between two-group of continuous variables was operated using the Student’s t-test or Mann-Whitney U test. The Chi-squared test was used for categorical variables. Correlations between variables were tested with Pearson Correlation. The cumulative HRV rates were performed using the Kaplan-Meier method and comparisons were tested with the log-rank test. Predictive factors for HRV were evaluated *via* the univariable and multivariable Cox proportional hazard analyses. Variables with p <0.10 in the univariable Cox proportional hazard analyses were used in multivariable analyses. Time-dependent receiver operator characteristic (ROC) curve analysis was conducted to investigate the predictive performance of anti-HBs and anti-HBc using the “timeROC” package in R (version 4.1.1, http://www.r-project.org). The optimal cut-off values of anti-HBs and anti-HBc were determined by ROC analysis. The cut-off value of the consolidation treatment duration was based on the previous study (Li et al., 2019). A P-value <0.05 was considered statistically significant. Analyses were performed using the SPSS version 20.0 (SPSS, Chicago, USA).

## 3 Results

### 3.1 Patient characteristics and HRV development

Clinical characteristics of 112 CHB patients who achieved PEG-IFN-induced HBsAg loss were presented in **Table 1**. The median age of total patients was 37.0 years (IQR, 31.0-43.0 years) and the majority of the patients were male (91/112, 81.3%). The median duration of PEG-IFN treatment and consolidation treatment was 48.0 weeks (48.0-60.0 weeks) and 24.0 weeks (12.0-30.0 weeks), respectively. The median follow-up duration was 12 months (5.3-32.5 months).

**Table 1:** Summary of 17 patients with HBsAg reversion after PEG-IFN therapy discontinuation.

Patient	Gender	Age (y)	Therapy	HBeAg status^a^	Anti-HBe status^a^	Anti-HBs (IU/L)^a^	Anti-HBc (s/co)^a^	Follow-up time (month)^c^	HBsAg (IU/mL)^b^	Anti-HBs (IU/L)^b^	HBV DNA (IU/mL)^b^	ALT (U/L)^b^
1	Male	37	PEG-IFN	Negative	Negative	<10	11.4	29	0.69	<10	<500	14
2	Male	51	PEG-IFN	Negative	Negative	142.3	11.3	48	250	<10	2.02E+07	225
3	Female	33	TDF+PEG-IFN	Negative	Positive	17.9	8.8	12	1.6	<10	<500	9
4	Male	46	TDF+PEG-IFN	Positive	Negative	16.2	5.4	4	0.33	<10	<500	53
5	Female	30	ETV+PEG-IFN	Positive	Negative	30.7	8.8	15	7.92	<10	<500	16
6	Male	46	TDF+PEG-IFN	Positive	Negative	186.7	9.3	24	0.25	<10	<500	29
7	Female	39	PEG-IFN	Negative	Positive	47.4	8.9	2	0.32	<10	<500	13
8	Male	39	TDF+PEG-IFN	Negative	Positive	14.1	9.3	10	0.08	<10	<500	16
9	Male	48	ETV+PEG-IFN	Negative	Negative	<10	7.5	4	0.08	<10	<500	23
10	Male	42	ADV+PEG-IFN	Negative	Positive	<10	8.1	31	0.26	<10	<500	29
11	Female	37	PEG-IFN	Positive	Negative	143.8	9.4	12	1.8	513.2	<500	10
12	Male	43	PEG-IFN	Negative	Positive	<10	8.1	3	0.14	<10	<500	31
13	Female	31	PEG-IFN	Negative	Positive	<10	8.9	3	0.75	<10	<500	19
14	Male	45	ADV+PEG-IFN	Negative	Positive	<10	7.1	1	2.34	<10	<500	226
15	Male	38	PEG-IFN	Negative	Positive	<10	9.5	1	0.22	<10	<500	27
16	Female	29	PEG-IFN	Negative	Positive	60.2	8.2	14	0.13	<10	<500	10
17	Male	40	PEG-IFN	Negative	Positive	<10	——	3	0.07	<10	<500	——

HBeAg, hepatitis B e antigen; HBsAg, hepatitis B surface antigen; Anti-HBs, hepatitis B surface antibody; Anti-HBc, hepatitis B core antibody; Anti-HBe, hepatitis B e antibody; ALT, alanine transaminase; PEG-IFN, pegylated interferon; ETV, entecavir; TDF, tenofovir; ADV, adefovir.

a At the end of PEG-IFN treatment.

b At the time of HBsAg reversion.

c Months from the date of the end of PEG-IFN treatment to the date of HBsAg reversion.

**Figure 2:**
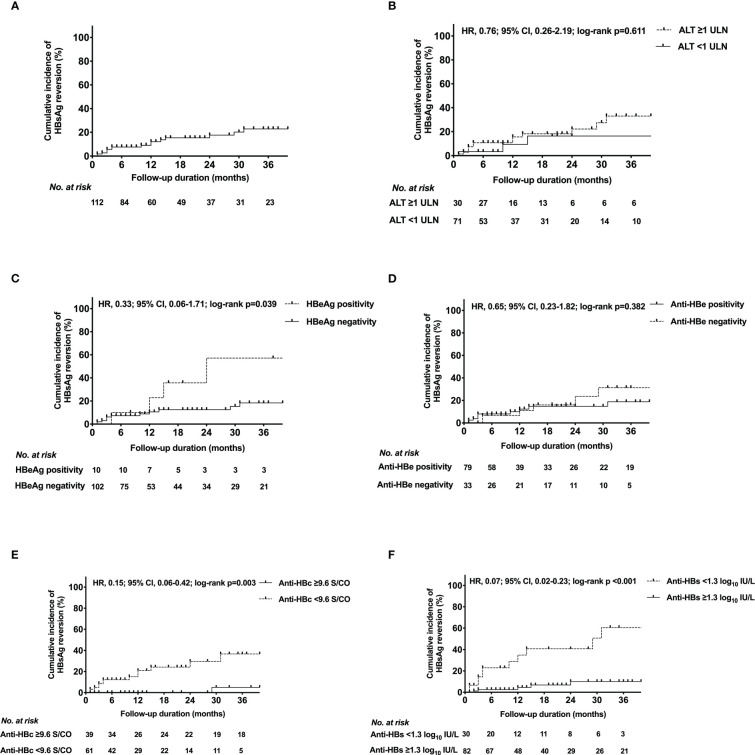
Kaplan-Meier estimates of cumulative incidence of HBsAg reversion in **(A)** the entire population, patients with different **(B)** ALT levels, **(C)** HBeAg status, **(D)** anti-HBe status, **(E)** anti-HBc level, **(F)** anti-HBs level at EOT. HBeAg, hepatitis B e antigen; HBsAg, hepatitis B surface antigen; Anti-HBs, hepatitis B surface antibody; Anti-HBc, hepatitis B core antibody; Anti-HBe, hepatitis B e antibody; ALT, alanine transaminase; ULN, upper limit of normal; HRV, hazard ratio; CI, confidence interval; EOT, end of PEG-IFN treatment.

17 patients developed HRV during the follow-up period (**Table 1**). The HRV peak was within 15 months (13 patients with HRV were observed). The overall cumulative incidence of HRV by Kaplan-Meier analysis was shown in **Figure 2A**. The 6-, 12-, 18-, 24-, 30-, 36-month cumulative incidence rates of HRV were 7.6%, 12.1%, 15.4%, 17.7%, 20.3%, 22.9% respectively.

### 3.2 Comparison of clinical characteristics between patients with CHL and HRV

Of the total patients, 95 (84.8%) remained HBsAg negativity during the follow-up period whereas 17 (15.2%) experienced HRV (**Table 2**). The positivity of anti-HBs at EOT was more frequent in the CHL group than in the HRV group (87.4% *vs*. 64.7%, p=0.047). During the follow-up period, the disappearance of anti-HBs more commonly occurred in the HRV group (p <0.001).

**Table 2:** Clinical characteristics of 112 CHB patients with HBsAg loss during and post PEG-IFN treatment.

	Total N=112	Confirmed HBsAg loss (CHL)^a^ N=95	HBsAg reversion (HRV)^b^ N=17	P value
Age (years)	37.0 (31.0-43.0)	36.0 (30.0-43.0)	39.0 (35.0-45.5)	0.355
Male, n (%)	91 (81.3%)	79 (83.2%)	12 (70.6%)	0.376
ALT (U/L)	36.0 (26.5-58.0)	36.5 (27.0-58.5)	33.0 (23.0-52.0)	0.516
HBeAg negativity, n (%)	98 (87.5%)	86 (90.5%)	13 (76.5%)	0.750
Therapeutic regimen, n (%)				0.636
PEG-IFN monotherapy	35 (31.2%)	29 (30.5%)	6 (35.3%)	
Add-on PEG-IFN^c^	46 (41.1%)	38 (40.0%)	8 (47.1%)	
Switch-to PEG-IFN^d^	31 (27.7%)	28 (29.5%)	3 (17.6%)	
Therapeutic medication, n (%)				0.393
PEG-IFN only	66 (58.9%)	57 (60%)	9 (52.9%)	
ETV+PEG-IFN	5 (4.5%)	3 (3.2%)	2 (11.8%)	
TDF+PEG-IFN	28 (25%)	24 (25.3%)	4 (23.5%)	
TAF+PEG-IFN	1 (0.9%)	1 (1.1%)	0 (0%)	
LAM+PEG-IFN	5 (4.5%)	5 (5.3%)	0 (0%)	
ADV+PEG-IFN	7 (6.3%)	5 (5.3%)	2 (11.8%)	
Platelet (×10^9^/L)	129.5 (103.8-171)	129.5 (104.0-163.8)	120.5 (90.0-181.5)	0.748
Albumin (g/L)	45.8 (45.0-48.7)	47.0 (45.0-49.0)	46.0 (44.0-47.8)	0.314
Bilirubin (μmol/L)	10.7 (8.0-15.4)	11.0 (8.1-16.1)	8.9 (8.0-13.0)	0.319
Positive anti-HBs at the time of HBsAg loss, n (%)	49 (43.8%)	42 (44.2%)	7 (41.2%)	0.816
Positive anti-HBs at EOT, n (%)	94 (83.9%)	83 (87.4%)	11 (64.7%)	0.047
Disappearance of anti-HBs during the follow-up period, n (%)	17 (15.2%)	8 (10.1%)	9 (81.8%)	<0.001
Positive anti-HBe, n (%)	78 (69.6%)	69 (73.4%)	9 (52.9%)	0.089
Anti-HBc (S/CO)	9.3 (8.1-10.1)	9.4 (8.2-10.1)	8.9 (8.1-9.4)	0.157
Undetectable HBV DNA	112 (100%)	95 (100%)	17 (100%)	>0.05
Consolidation treatment duration (weeks)^e^	24.0 (12.0-30.0)	24.0 (12.0-36.0)	12.0 (4.0-24.0)	0.177
PEG-IFN duration (weeks)	48.0 (48.0-72.0)	48.0 (48.0-72.0)	48.0 (48.0-80.0)	0.517
Follow-up duration (months)^f^	12.0 (5.3-32.5)	14.0 (6.0-35.0)	10.0 (3.0-19.5)	0.061

ALT, alanine transaminase; HBsAg, hepatitis B surface antigen; HBeAg, hepatitis B e antigen; Anti-HBs, hepatitis B surface antibody; Anti-HBc, hepatitis B core antibody; Anti-HBe, hepatitis B e antibody; PEG-IFN, pegylated interferon; ETV, entecavir; TDF, tenofovir; TAF, tenofovir alafenamide; LAM, lamivudine; ADV, adefovir; EOT, end of PEG-IFN treatment.

a Two negative HBsAg results (<0.05 IU/mL) at least 6 months apart.

b Reappearance of HBsAg after HBsAg loss.

c Combination therapy after at least 48 weeks of nucleot(s)ide therapy.

d PEG-IFN monotherapy in patients who received NAs for at least 48 weeks.

e Weeks from the date of HBsAg loss to the date of the end of PEG-IFN treatment.

f Months from the date of the end of PEG-IFN treatment to the date of HR or the last follow-up visit.

### 3.3 Cumulative incidence of HRV based on HBV markers and ALT level at EOT

The cumulative incidence curves of HRV for each HBV marker and ALT level at EOT were presented in **Figures 2B–F**. Based on the ROC analysis, the optimal cut-off values of anti-HBs and anti-HBc at EOT were 1.3 log_10_IU/L and 9.6 S/CO, respectively (**Supplementary Figure 1**). The incidence of HRV differed significantly between the following subgroups: Anti-HBc ≥9.6 S/CO *vs*. <9.6 S/CO (p=0.003); HBeAg positivity *vs*. negativity (p=0.039); Anti-HBs ≥1.3 log_10_IU/L *vs*. <1.3 log_10_IU/L (p<0.001). Conversely, the incidence of HRV did not differ between anti-HBe positivity *vs*. negativity (p=0.382); ALT ≥1 upper limit of normal (ULN) *vs*. <1 ULN (p=0.611).

### 3.4 Time-dependent ROC analysis for incidence of HRV

**Figure 3:**
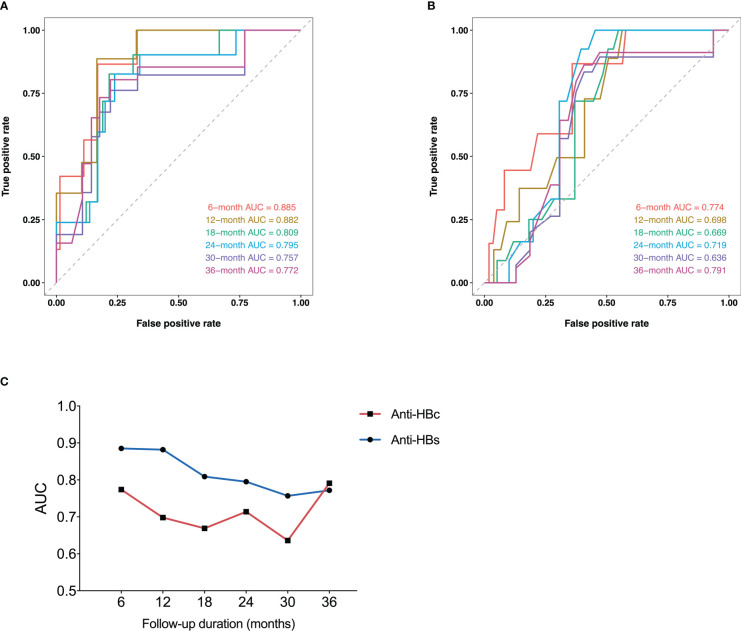
Time-dependent ROC curves of anti-HBs **(A)** and anti-HBc **(B)** at EOT for HRV incidence at 6, 12, 18, 24, 30, and 36 months after the start of follow-up. Plots of AUCs of anti-HBs and anti-HBc at the same time point as above **(C)**. ROC, receiver operating characteristic curve; Anti-HBs, hepatitis B surface antibody; Anti-HBc, hepatitis B core antibody; HRV, HBsAg reversion; AUC, area under the receiver operating characteristic curve; EOT, end of PEG-IFN treatment.

The respective ROC curves of the anti-HBs level and anti-HBc at EOT for the incidence of HRV at 6, 12, 18, 24, 30, and 36 months after the start of follow-up were shown in **Figures 3A, B** respectively. The AUCs for anti-HBs at the above time points were 0.885, 0.882, 0.809, 0.795, 0.757, 0.772, respectively. The AUCs for anti-HBc at the above time points were 0.774, 0.698, 0.669, 0.714, 0.636, 0.791, respectively.

The plots of AUCs of anti-HBs and anti-HBc for the incidence of HRV from 6 to 36 months after the start of follow-up were shown in **Figure 3C**. The predictive power of anti-HBs was superior to that of anti-HBc from 6 to 36 months in general.

### 3.5 Predictive factors of HRV

Based on the multivariable analysis, anti-HBs ≥1.3 log_10_IU/L at EOT (hazard ratio (HR), 0.148; 95% confidence interval (CI), 0.044-0.502; p=0.002) and HBeAg negativity at EOT (HR, 0.183; 95%CI, 0.052-0.639, p=0.008) were independently associated with lower incidence of HR (**Table 3**).

**Table 3:** Predictive factors of HBsAg reversion.

	Univariate analysis			Multivariable analysis^a^		
Variables	HR	95% CI	p value	HR	95% CI	p value
Age ≥36.5 years	1.761	0.551 to 5.626	0.340			
Male	0.460	0.161 to 1.314	0.147			
HBeAg negativity at EOT	0.323	0.104 to 1.007	0.051	0.183	0.052 to 0.639	0.008
ALT ≥1 ULN at EOT	0.562	0.182 to 1.711	0.316			
Consolidation treatment ≥12 weeks^b^	0.529	0.195 to 1.439	0.212			
Anti-HBs ≥1.3 log_10_IU/L at EOT	0.214	0.078 to 0.583	0.003	0.148	0.044 to 0.502	0.002
Anti-HBe positivity at EOT	0.652	0.248 to 1.718	0.387			
Anti-HBc ≥9.6 S/CO at EOT	0.134	0.029 to 0.622	0.010	0.328	0.064 to 1.682	0.181
NA-experienced	0.958	0.353 to 2.602	0.933			

ALT, alanine transaminase; HBeAg, hepatitis B e antigen; Anti-HBs, hepatitis B surface antibody; Anti-HBc, hepatitis B core antibody; Anti-HBe, hepatitis B e antibody; EOT, end of PEG-IFN treatment; NA, nucleos(t)ide analogue; HR, hazard ratio; CI, confidence interval; ULN, upper limit of normal.

a Multivariable analysis including variables with p<0.10 at univariate analysis.

b Weeks from the date of HBsAg loss to the date of the end of PEG-IFN treatment.

**Figure 4:**
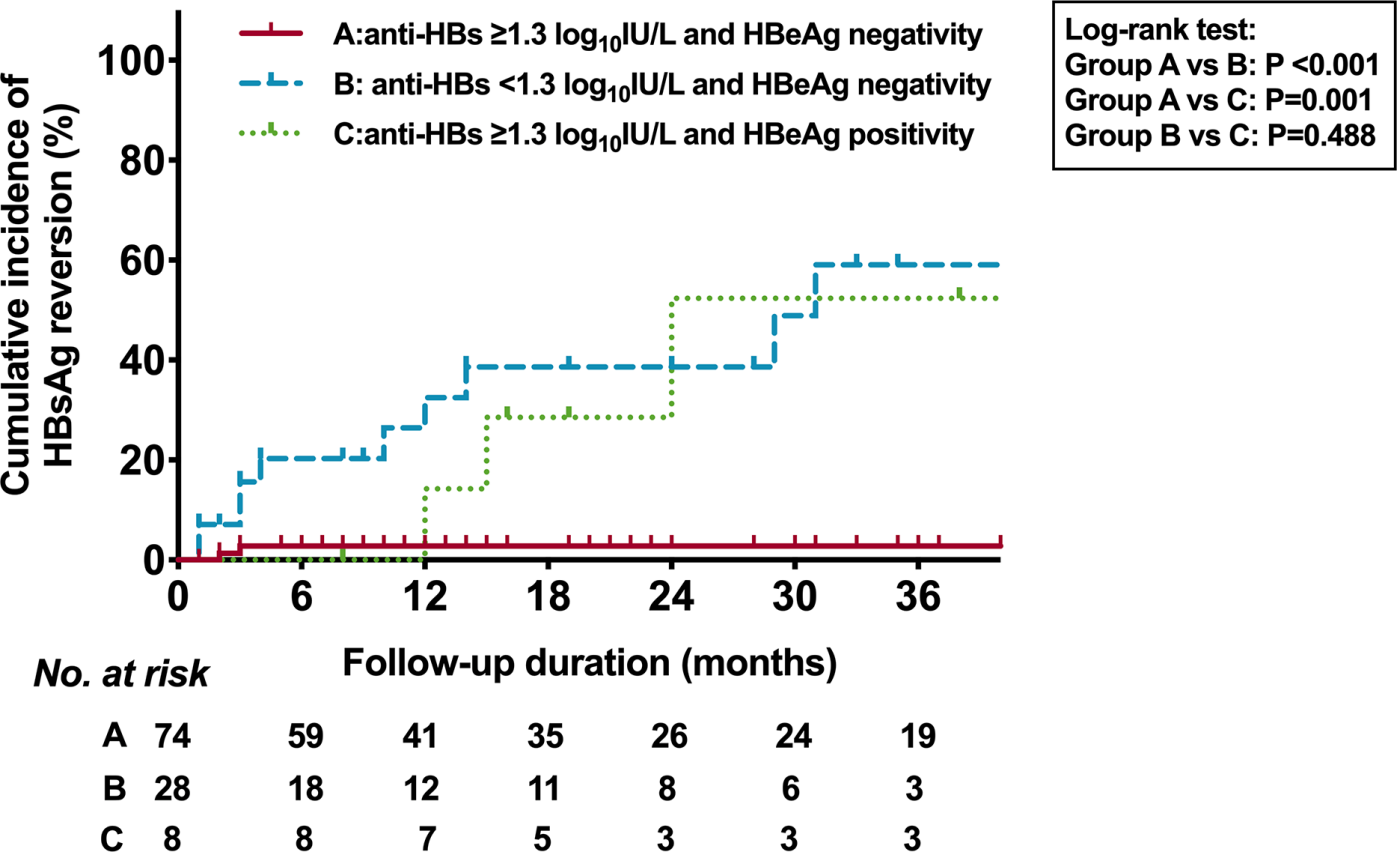
Kaplan-Meier estimates of cumulative incidence of HBsAg reversion based on HBeAg status and anti-HBs of 1.3 log10IU/L at EOT. HBsAg, hepatitis B surface antigen; HBeAg, hepatitis B e antigen; Anti-HBs, hepatitis B surface antibody; EOT, end of PEG-IFN treatment.

**Figure 5:**
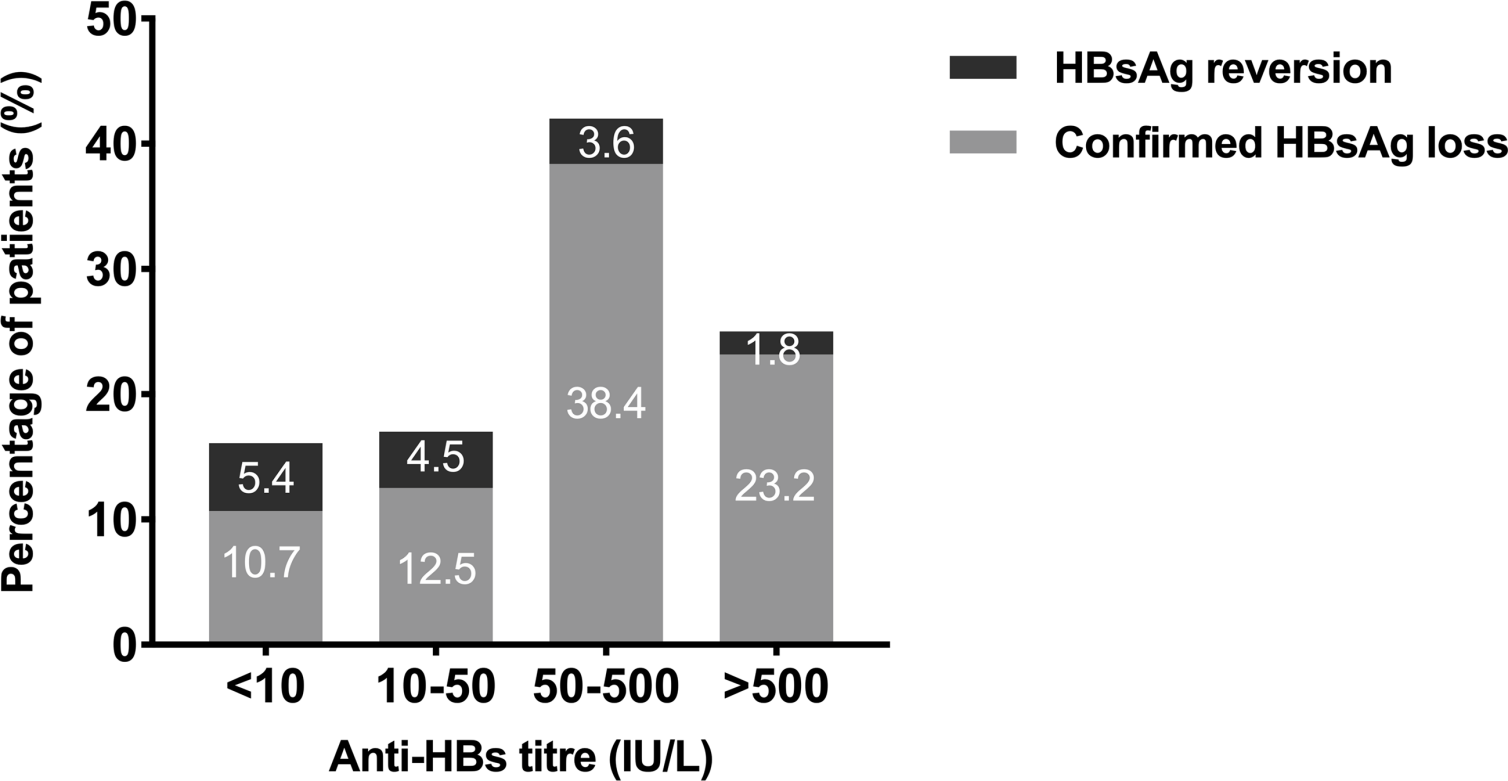
Percentage of patients with HRV according to different anti-HBs levels at EOT. HRV, HBsAg reversion; Anti-HBs, hepatitis B surface antibody; EOT, end of PEG-IFN treatment.

We then analyzed the association between the cumulative incidence of HRV and the combination of HBeAg status and anti-HBs level at EOT (**Figure 4**). Among the patients with HBeAg negativity at EOT, the 36-month cumulative incidence rates of HRV in patients with anti-HBs ≥1.3 log_10_IU/L and <1.3 log_10_IU/L were 2.8% and 59.1% (P <0.001), respectively. Among the patients with anti-HBs ≥1.3 log_10_IU/L at EOT, the 36-month cumulative incidence rates of HRV in patients with HBeAg negativity and positivity were 2.8% and 52.4% (P=0.001), respectively. Moreover, the percentage of patients with HRV or CHL based on the different anti-HBs levels at EOT was shown in **Figure 5**.

### 3.6 Cross-sectional analysis of serum HBV RNA level

**Figure 6:**
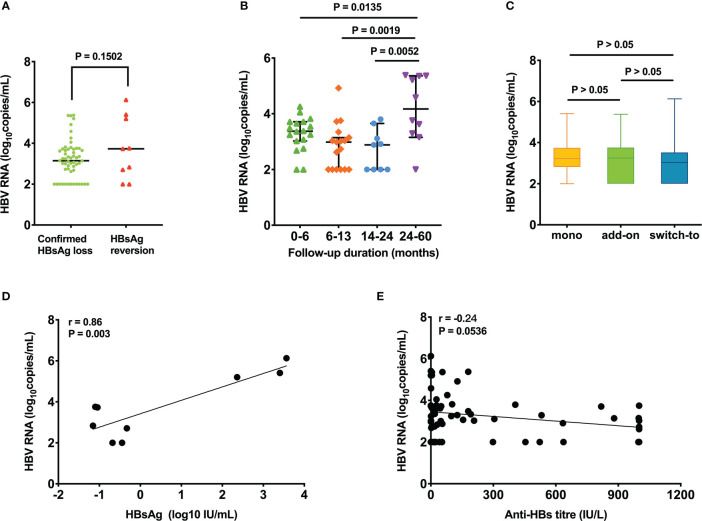
Cross-sectional analysis of serum HBV RNA level. Comparison of HBV RNA level in patients between **(A)** the CHL and HRV group, **(B)** different follow-up periods, and **(C)** different therapeutic regimens. Correlation between serum HBV RNA and **(D)** HBsAg level in patients with HRV, **(E)** anti-HBs level in the entire population. CHL, confirmed HBsAg loss; HRV, HBsAg reversion; HBsAg, hepatitis B surface antigen; Anti-HBs, hepatitis B surface antibody. Mono, pegylated interferon (PEG-IFN) monotherapy in patients who did not receive nucleot(s)ide therapy ever; Add-on, combination therapy after at least 48 weeks of nucleot(s)ide therapy; Switch-to, PEG-IFN monotherapy in patients who received NAs for at least 48 weeks.

To understand the serum HBV RNA level in patients with CHL and HRV, a cross-sectional analysis of serum HBV RNA levels was performed in samples of 64 patients (**Supplementary Table 1**). There were 55 patients with CHL and 9 patients with HRV. No significant difference between the CHL and HRV groups (p=0.1502) (**Figure 6A**). Among the patients with CHL, the HBV RNA levels in patients at 24-60 months after the start of follow-up was significantly higher than that in patients at 0-6 months (p=0.0135), 6-13 months (p=0.0019), and 14-24 months (p=0.0052) (**Figure 6B**). Different therapeutic regimens including “PEG-IFN monotherapy”, “Add-on”, and “Switch-to” showed no difference in HBV RNA level (**Figure 6C**). HBV RNA levels were strongly correlated with HBsAg levels in patients with HRV (r=0.86, p=0.003) (**Figure 6D**). Anti-HBs titers of 64 patients were not correlated with HBV RNA levels (r=-0.24, p=0.0536) (**Figure 6E**).

## 4 Discussion

This retrospective cohort study mainly identified predictive factors associated with HRV in CHB patients who achieved PEG-IFN-induced HBsAg loss. Univariable analysis using the log-rank test showed that the status or level of HBeAg, anti-HBs, and anti-HBc were significantly associated with the incidence of HRV. The predictive power of anti-HBs was superior to that of anti-HBc during the follow-up period (from 6 to 36 months) in general. Furthermore, multivariable Cox proportional hazard analysis showed that anti-HBs ≥1.3 log_10_IU/L, and HBeAg negativity at EOT were independently associated with lower incidence of HRV.

The role of the anti-HBs in the durability of HBsAg loss remains controversial (Paul et al., 2017; Yip et al., 2017; Li et al., 2019; Alawad et al., 2020; Wu et al., 2020; Song et al., 2021; Huang et al., 2022). For patients with PEG-IFN-induced HBsAg loss, Wu et al. reported in their retrospective cohort study that the average HRV time for anti-HBs ≥100 IU/L was longer than that for anti-HBs <100 IU/L (107 weeks *vs*. 41 weeks, P <0.001) (Wu et al., 2020). Li et al. reported that consolidation treatment ≥12 weeks and high anti-HBs levels were strong predictors of HRV in HBeAg-negative patients (Li et al., 2019). Huang et al. reported that HBcrAg <4 log_10_U/mL and anti-HBs >2 log_10_IU/L could predict the sustained functional cure (Huang et al., 2022). However, for patients with spontaneous HBsAg loss and NAs-induced HBsAg loss, Yip et al. reported that anti-HBs negativity at EOT was not associated with HRV (Yip et al., 2017). In the present study, we found that not only was anti-HBs disappearance more common in the HRV group but also the anti-HBs levels at EOT were associated with HRV in univariable and multivariable analyses. The cut-off anti-HBs level at EOT for predicting HRV was 1.3 log_10_IU/L with a sensitivity of 0.65 and a specificity of 0.80 in our cohort. In addition, the optimal anti-HBs titer for predicting sustained functional cure remained unclear. We also used the cut-off anti-HBs level (2 log_10_IU/L) based on previous studies (Wu et al., 2020; Huang et al., 2022). The incidence of HRV in patients with anti-HBs ≥2 log_10_IU/L was significantly lower than that in patients with anti-HBs <2 log_10_IU/L (p=0.002) (**Supplementary Figure 2**). The multivariable analysis also showed that anti-HBs ≥2 log_10_IU/L and HBeAg negativity at EOT were significantly associated with HRV (**Supplementary Table 2**). Furthermore, to our knowledge, no previous studies have used time-dependent ROC analysis to evaluate anti-HBs or anti-HBc in terms of their association with the incidence of HRV after PEG-IFN discontinuation. We used this analysis to show that the anti-HBs level was mainly superior to the anti-HBc level regarding the predictive power for HRV over 36 months. Collectively, our results highlighted the clinical significance of anti-HBs levels at EOT in predicting HRV. However, the underlying mechanisms remain unclear. In contrast to the constant production of anti-HBc, the anti-HBs production displays functional defects in CHB patients. In a study on the HBV humoral immunity, researchers found that HBcAg-specific B cells exhibited higher frequency and more mature phenotype than HBsAg-specific B cells (Le Bert et al., 2020). The appearance of anti-HBs may indicate sustained and profound anti-HBV immunity elicited by PEG-IFN treatment, NAs treatment, or other factors, which need to be examined in great detail.

In addition to anti-HBs levels, patients with HBeAg positivity at EOT showed a significantly higher incidence of HRV. Moreover, HBeAg status at EOT was also identified as an independent predictor of HRV by multivariable analysis. However, a recent prospective study suggested that HBeAg at EOT was not indispensable for maintaining PEG-IFN-induced HBsAg loss (Huang et al., 2022). The inconsistency may partly be ascribed to the following differences in our patient cohort: (a) The cut-off value of HBeAg was based on negativity (<1 S/CO) and positivity (>1 S/CO) rather than the Youden’s index; (b) Our patient cohort was relatively large; and (c) HBeAg was still positive in a small proportion of patients at EOT. Intriguingly, the same study reported that hepatitis B core-related antigen (HBcrAg) <4 log_10_U/mL was one of the significant protectors from HRV during the off-treatment follow-up. Furthermore, the HBcrAg level was positively correlated with the HBeAg level at EOT (Huang et al., 2022). Further studies with a larger population are warranted to clarify the role of HBeAg and HBcrAg in the durability of HBsAg loss.

Consolidation treatment duration has been reported to be associated with HRV following therapy discontinuation (Yip et al., 2017; Li et al., 2019). Conversely, the duration of consolidation treatment displayed no significant difference between the CHL and the HRV group in this study. We further analyzed the incidence of HRV using 12 weeks as the cut-off value, the incidence of HRV did not differ between patients with consolidation treatment <12 weeks *vs*. ≥12 weeks (**Supplementary Figure 3**).

Serum HBV RNA is regarded as an alternative biomarker for cccDNA activity (Wang et al., 2016; Gao et al., 2017; Huang et al., 2018). In this regard, several studies have suggested that HBV RNA can be used to predict virologic and clinical relapse after discontinuation of antiviral therapy (Wang et al., 2016; Carey et al., 2020; Fan et al., 2020). Nonetheless, HBV RNA alone may not be insufficient to assess sustained response, researchers found that many patients experiencing HBV RNA decline during PEG-IFN treatment did not achieve HBsAg and/or HBcrAg decline (Brakenhoff et al., 2021). In the present study, there were significant associations between higher HBV RNA levels and HBsAg levels in patients with HRV, in line with previous studies (Lin et al., 2020; Ghany et al., 2021). Thus, low HBsAg levels in the HRV group may account for no significant difference between the CHL and HRV groups. We also found that there was a trend that anti-HBs level in patients with CHL was negatively correlated with HBV RNA. For patients with CHL, HBV RNA levels did not differ across different therapeutic regimens, consistent with the observation that there was no statistical difference in the therapeutic regimens between the CHL and HRV groups. Similarly, Pan et al. also reported that add-on interferon treatment and interferon monotherapy exhibited equivalent efficacy in sustained functional cure (Pan et al., 2021). Additionally, HBV RNA at 24-60 months had higher HBV RNA levels than in other periods. The exact reasons for the discrepancy are unclear. Longitudinal studies on HBV RNA levels after PEG-IFN discontinuation are warranted to validate these above results.

This study has several limitations. First, this was a retrospective study and not all patients were regularly monitored. Thus, we cannot exclude the possibility of transient HRV between adjacent visits. Second, genotype data cannot be obtained in this study. Whether the genotype plays a role in the durability of HBsAg loss has not been determined. Third, as another novel biomarker, HBcrAg may be associated with HRV and could not be analyzed because serum samples were not available (Huang et al., 2022). Fourth, serum HBV RNA was investigated in a cross-sectional analysis. We could not analyze the longitudinal changes of this novel biomarker, especially after the PEG-IFN discontinuation in patients with CHL.

In conclusion, HBeAg status (negativity) and anti-HBs level (≥1.3 log_10_IU/L) at EOT were associated with durability of PEG-IFN-induced HBsAg loss. These findings should be further investigated on a larger sample size.

## Data availability statement

The raw data supporting the conclusions of this article will be made available by the authors, without undue reservation.

## Ethics statement

The studies involving human participants were reviewed and approved by Huashan Hospital of Fudan University(KY2015-212). The patients/participants provided their written informed consent to participate in this study.

## Author contributions

All authors contributed equally to the concept and preparation of the manuscript. YFG and JZ completed the final preparation and editing of the manuscript. YFG analyzed the data and created the figures and tables. All authors contributed to the article and approved the submitted version.

## References

[B1] Alawad A. S. Auh S. Suarez D. Ghany M. G. (2020). Durability of spontaneous and treatment-related loss of hepatitis b s antigen. Clin. Gastroenterol. Hepatol. 18 (3), 700–709.e3. doi: 10.1016/j.cgh.2019.07.018 31323381 PMC6962568

[B2] Brakenhoff S. M. de Man R. A. Boonstra A. van Campenhout M. J. H. de Knegt R. J. van Bömmel F.. (2021). Hepatitis b virus RNA decline without concomitant viral antigen decrease is associated with a low probability of sustained response and hepatitis b surface antigen loss. Alimentary Pharmacol. Ther. 53 (2), 314–320. doi: 10.1111/apt.16172 PMC7839551 33222190

[B3] Carey I. Gersch J. Wang B. Moigboi C. Kuhns M. Cloherty G.. (2020). Pregenomic HBV RNA and hepatitis b core-related antigen predict outcomes in hepatitis b e antigen-negative chronic hepatitis b patients suppressed on Nucleos(T)ide analogue therapy. Hepatol. (Baltimore Md). 72 (1), 42–57. doi: 10.1002/hep.31026 31701544

[B4] European Association for the Study of the Liver. (2017). EASL 2017 clinical practice guidelines on the management of hepatitis B virus infection. J. Hepatol. 67 (2), 370–398. doi: 10.1016/j.jhep.2017.03.021 28427875

[B5] Fan R. Zhou B. Xu M. Tan D. Niu J. Wang H.. (2020). Association between negative results from tests for HBV DNA and RNA and durability of response after discontinuation of nucles(t)ide analogue therapy. Clin. Gastroenterol. Hepatol.: Off. Clin. Pract. J. Am. Gastroenterological Assoc. 18 (3), 719–727.e7. doi: 10.1016/j.cgh.2019.07.046 31362119

[B6] Gao Y. Li Y. Meng Q. Zhang Z. Zhao P. Shang Q.. (2017). Serum Hepatitis B Virus DNA, RNA, And HBsAg: Which correlated better with intrahepatic covalently closed circular DNA before and after nucleos(t)ide analogue treatment? J. Clin. Microbiol. 55 (10), 2972–2982. doi: 10.1128/JCM.00760-17 28747369 PMC5625383

[B7] Ghany M. G. King W. C. Lisker-Melman M. Lok A. S. F. Terrault N. Janssen H. L. A.. (2021). Comparison of HBV RNA and hepatitis b core related antigen with conventional HBV markers among untreated adults with chronic hepatitis b in north America. Hepatol. (Baltimore Md). 74 (5), 2395–2409. doi: 10.1002/hep.32018 PMC8895675 34133774

[B8] Huang H. Wang J. Li W. Chen R. Chen X. Zhang F.. (2018). Serum HBV DNA plus RNA shows superiority in reflecting the activity of intrahepatic cccDNA in treatment-naïve HBV-infected individuals. J. Clin. Virol. 99-100, 71–78. doi: 10.1016/j.jcv.2017.12.016 29353073

[B9] Huang D. Wu D. Wang P. Wang Y. Yuan W. Hu D.. (2022). End-of-treatment HBcrAg and HBsAb levels identify durable functional cure after peg-IFN-based therapy in patients with CHB. J. Hepatol. 77 (1), 42–54. doi: 10.1016/j.jhep.2022.01.021 35149125

[B10] Le Bert N. Salimzadeh L. Gill U. S. Dutertre C.-A. Facchetti F. Tan A.. (2020). Comparative characterization of b cells specific for HBV nucleocapsid and envelope proteins in patients with chronic hepatitis b. J. Hepatol. 72 (1), 34–44. doi: 10.1016/j.jhep.2019.07.015 31348999

[B11] Li M.-H. Yi W. Zhang L. Lu Y. Lu H.-H. Shen G.. (2019). Predictors of sustained functional cure in hepatitis b envelope antigen-negative patients achieving hepatitis b surface antigen seroclearance with interferon-alpha-based therapy. J. Viral Hepat. 26 Suppl 1, 32–41. doi: 10.1111/jvh.13151 31380582

[B12] Lin N. Ye A. Lin J. Liu C. Huang J. Fu Y.. (2020). Diagnostic value of detection of pregenomic RNA in sera of hepatitis b virus-infected patients with different clinical outcomes. J. Clin. Microbiol. 58 (2), e01275–19. doi: 10.1128/JCM.01275-19 31723011 PMC6989074

[B13] Marcellin P. Ahn S. H. Ma X. Caruntu F. A. Tak W. Y. Elkashab M.. (2016). Combination of tenofovir disoproxil fumarate and peginterferon α-2a increases loss of hepatitis b surface antigen in patients with chronic hepatitis b. Gastroenterology 150 (1), 134–144.e10. doi: 10.1053/j.gastro.2015.09.043 26453773

[B14] Pan C. Q. Li M.-H. Yi W. Zhang L. Lu Y. Hao H.-X.. (2021). Outcome of Chinese patients with hepatitis b at 96 weeks after functional cure with IFN versus combination regimens. Liver International: Off. J. Int. Assoc. For Stud. Liver 41 (7), 1498–1508. doi: 10.1111/liv.14801 33486874

[B15] Paul S. Dickstein A. Saxena A. Terrin N. Viveiros K. Balk E. M.. (2017). Role of surface antibody in hepatitis b reactivation in patients with resolved infection and hematologic malignancy: A meta-analysis. Hepatol. (Baltimore Md). 66 (2), 379–388. doi: 10.1002/hep.29082 PMC6485929 28128861

[B16] Sarin S. K. Kumar M. Lau G. K. Abbas Z. Chan H. L. Y. Chen C. J.. (2016). Asian-Pacific clinical practice guidelines on the management of hepatitis b: A 2015 update. Hepatol. Int. 10 (1), 1–98. doi: 10.1007/s12072-015-9675-4 PMC4722087 26563120

[B17] Song A. Wang X. Lu J. Jin Y. Ma L. Hu Z.. (2021). Durability of hepatitis b surface antigen seroclearance and subsequent risk for hepatocellular carcinoma: A meta-analysis. J. Viral Hepatitis 28 (4), 601–612. doi: 10.1111/jvh.13471 PMC7986681 33455067

[B18] Terrault N. A. Bzowej N. H. Chang K.-M. Hwang J. P. Jonas M. M. Murad M. H. (2016). AASLD guidelines for treatment of chronic hepatitis b. Hepatol. (Baltimore Md). 63 (1), 261–283. doi: 10.1002/hep.28156 PMC5987259 26566064

[B19] Tout I. Lampertico P. Berg T. Asselah T. (2021). Perspectives on stopping nucleos(t)ide analogues therapy in patients with chronic hepatitis b. Antiviral Res. 185, 104992. doi: 10.1016/j.antiviral.2020.104992 33279523

[B20] Wang J. Shen T. Huang X. Kumar G. R. Chen X. Zeng Z.. (2016). Serum hepatitis b virus RNA is encapsidated pregenome RNA that may be associated with persistence of viral infection and rebound. J. Hepatol. 65 (4), 700–710. doi: 10.1016/j.jhep.2016.05.029 27245431

[B21] WHO (2017) Global hepatitis report. Available at: https://www.who.int/publications/i/item/9789241565455.

[B22] Wu Y. Liu Y. Lu J. Cao Z. Jin Y. Ma L.. (2020). Durability of interferon-induced hepatitis b surface antigen seroclearance. Clin. Gastroenterol. Hepatol. 18 (2), 514–516.e2. doi: 10.1016/j.cgh.2019.04.020 30981007

[B23] Wu D. Yan W. Tan D. Peng S. Chen Y. Jiang J.. (2020). Combination of NA, PEG-IFN alpha-2b and GM-CSF enhanced hbsab production in NA experienced CHB patients (the anchor a study): an interim analysis. J. Hepatol. 73, S860. doi: 10.1016/S0168-8278(20)32161-9

[B24] Yip T. C.-F. Wong G. L.-H. Wong V. W.-S. Tse Y.-K. Lui G. C.-Y. Lam K. L.-Y.. (2017). Durability of hepatitis b surface antigen seroclearance in untreated and nucleos(t)ide analogue-treated patients. J. Hepatol, S0168-8278(17)32332-2. doi: 10.1016/j.jhep.2017.09.018 28989093

