# Supplementary material for: End-of-treatment anti-HBs levels and HBeAg status identify durability of HBsAg loss after PEG-IFN discontinuation
Source: Front Cell Infect Microbiol. 2023 Feb 24;13:1120300. doi: 10.3389/fcimb.2023.1120300 (PMC9998526; doi:10.3389/fcimb.2023.1120300)
Supplement: Supplementary file 1 [file DataSheet_1.pdf]

Supplementary Material

End-of-treatment anti-HBs levels and HBeAg status identify durability of HBsAg loss after PEG-IFN discontinuation

Yifei Guo [1], Jiajia Han [1], Yao Zhang [1], Jingjing He[1], Shiqi Chen[1], Yue Guo[1], Yanxue Lin[1], Fahong Li[1], Feifei Yang[1], Zhongliang Shen[1], Richeng Mao[1], Haoxiang Zhu[1*], Jiming Zhang[1,2*]

[1]Department of Infectious Diseases, Shanghai Key Laboratory of Infectious Diseases and Biosafety Emergency Response, Shanghai Institute of Infectious Diseases and Biosecurity, National Medical Center for Infectious Diseases, Huashan Hospital, Fudan University, Shanghai, China

[2]Key Laboratory of Medical Molecular Virology (MOE/NHC/CAMS), Shanghai Frontiers Science Center of Pathogenic Microorganisms and Infection, School of Basic Medical Sciences, Shanghai Medical College, Fudan University, Shanghai, China

*** Correspondence:**
Haoxiang Zhu
haoxiangzhu2015@163.com

Jiming Zhang

jmzhang@fudan.edu.cn

Supplementary Figure legends

Supplementary Fig. 1. ROC curves of diagnostic performance of anti-HBs and anti-HBc at EOT for predicting HRV after PEG-IFN discontinuation. ROC, receiver operating characteristic curve; Anti-HBs, hepatitis B surface antibody; Anti-HBc, hepatitis B core antibody; PEG-IFN, pegylated interferon; EOT, end of PEG-IFN treatment; HRV, HBsAg reversion.

Supplementary Fig. 2. Kaplan-Meier estimates of cumulative incidence of HBsAg reversion based on anti-HBs of 2 log10IU/L at EOT. HBsAg, hepatitis B surface antigen; Anti-HBs, hepatitis B surface antibody; EOT, end of PEG-IFN treatment; HR, hazard ratio; CI, confidence interval.

Supplementary Fig. 3. Kaplan-Meier estimates of cumulative incidence of HBsAg reversion based on the consolidation duration of 12 weeks. HBsAg, hepatitis B surface antigen; HR, hazard ratio; CI, confidence interval.

Supplementary Table 1. Patient characteristics (N=64)

Age (years)	40.0 (32.0-45.8)
Male, n (%)	55 (85.9%)
HBsAg reversion, n (%)	9 (14.1%)
ALT (U/L)	31.0 (19.0-51.3)
HBeAg negativity, n (%)	55 (85.9%)
Platelet ($\times 10^9$/L)	178.5 (122.0-231.3)
Therapeutic regimen, n (%)	
PEG-IFN monotherapy	20 (31.3%)
Add-on PEG-IFN[a]	35 (54.7%)
Switch-to PEG-IFN[b]	9 (14.1%)
Anti-HBs positivity, n (%)	46 (71.9%)
Anti-HBs (IU/L)	127.2 (38.1-557.4)
Anti-HBc (S/CO)	8.6 (7.9-9.2)
HBV RNA (log_{10}copies/mL)	3.2 (2.6-3.7)
Follow-up duration (months)	12.0 (3.0-18.0)

ALT, alanine transaminase; HBsAg, hepatitis B surface antigen; HBeAg, hepatitis B e antigen; Anti-HBs, hepatitis B surface antibody; Anti-HBc, hepatitis B core antibody; PEG-IFN, pegylated interferon.

a Combination therapy after at least 48 weeks of nucleot(s)ide therapy.

b PEG-IFN monotherapy in patients who received NAs for at least 48 weeks.

Supplementary Table 2. Predictive factors of HBsAg reversion

	Univariate analysis			Multivariable analysis[a]		
Variables	HR	95% CI	p value	HR	95% CI	p value
Age ≥36.5 years	1.761	0.551 to 5.626	0.340			
Male	0.460	0.161 to 1.314	0.147			
HBeAg negativity at EOT	0.323	0.104 to 1.007	0.051	0.225	0.066 to 0.766	0.017
ALT ≥1 ULN at EOT	0.562	0.182 to 1.711	0.316			
Consolidation treatment ≥12 weeks[b]	0.529	0.195 to 1.439	0.212			
Anti-HBs ≥2 log_{10}IU/L at EOT	0.184	0.053 to 0.640	0.008	0.255	0.067 to 0.971	0.045
Anti-HBe positivity at EOT	0.652	0.248 to 1.718	0.387			
Anti-HBc ≥9.6 S/CO at EOT	0.134	0.029 to 0.622	0.010	0.232	0.047 to 1.142	0.072
NA-experienced	0.958	0.353 to 2.602	0.933			

ALT, alanine transaminase; HBeAg, hepatitis B e antigen; Anti-HBs, hepatitis B surface antibody; Anti-HBc, hepatitis B core antibody; Anti-HBe, hepatitis B e antibody; EOT, end of PEG-IFN treatment; NA, nucleos(t)ide analogue; HR, hazard ratio; CI, confidence interval; ULN, upper limit of normal.

a Multivariable analysis including variables with $p<0.10$ at univariate analysis.

b Weeks from the date of HBsAg loss to the date of the end of PEG-IFN treatment.
