# Supplementary figures and images for: End-of-treatment anti-HBs levels and HBeAg status identify durability of HBsAg loss after PEG-IFN discontinuation
Source: Front Cell Infect Microbiol. 2023 Feb 24;13:1120300. doi: 10.3389/fcimb.2023.1120300 (PMC9998526; doi:10.3389/fcimb.2023.1120300)

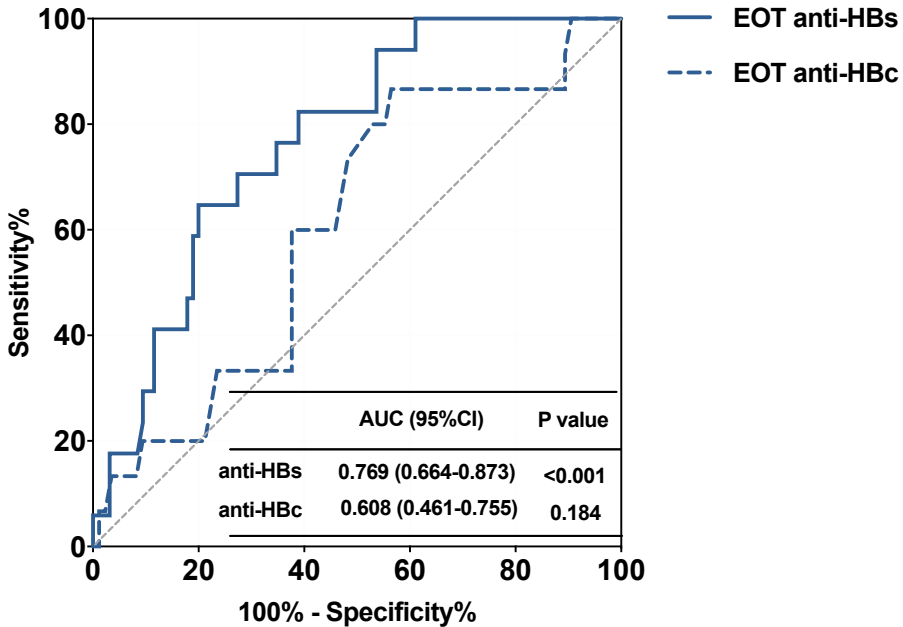

Supplement: Supplementary file 2 [file DataSheet_2.pdf]

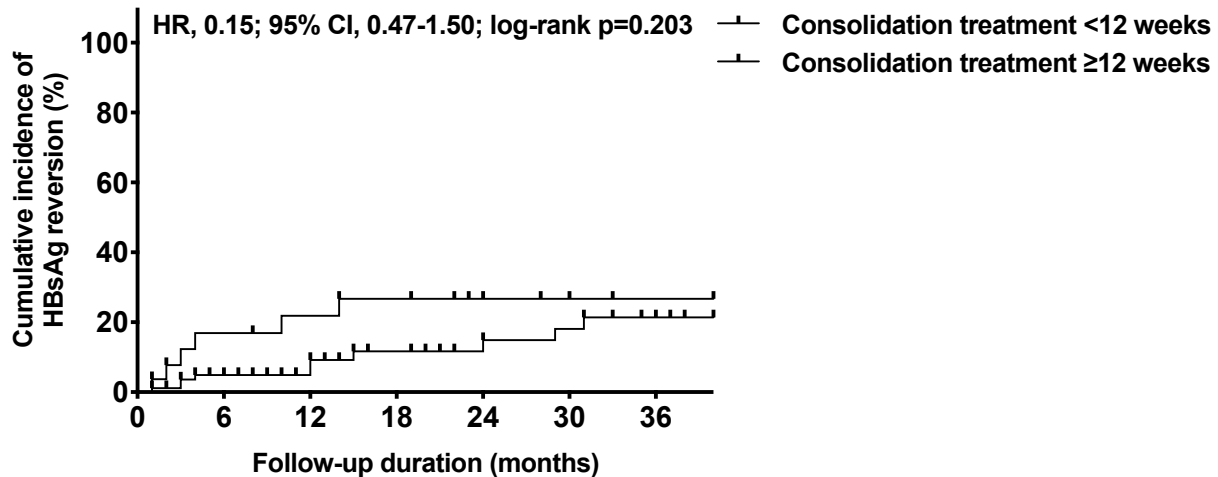

*No. at risk*

|                                   |    |    |    |    |    |    |    |
|-----------------------------------|----|----|----|----|----|----|----|
| Consolidation treatment <12 weeks | 27 | 19 | 17 | 16 | 9  | 6  | 5  |
| Consolidation treatment ≥12 weeks | 85 | 66 | 44 | 35 | 28 | 26 | 20 |

Supplement: Supplementary file 4 [file DataSheet_4.pdf]
