# Supplementary material for: End-of-treatment anti-HBs levels and HBeAg status identify durability of HBsAg loss after PEG-IFN discontinuation
Source: Front Cell Infect Microbiol. 2023 Feb 24;13:1120300. doi: 10.3389/fcimb.2023.1120300 (PMC9998526; doi:10.3389/fcimb.2023.1120300)
Supplement: Supplementary file 3 [file DataSheet_3.pdf]

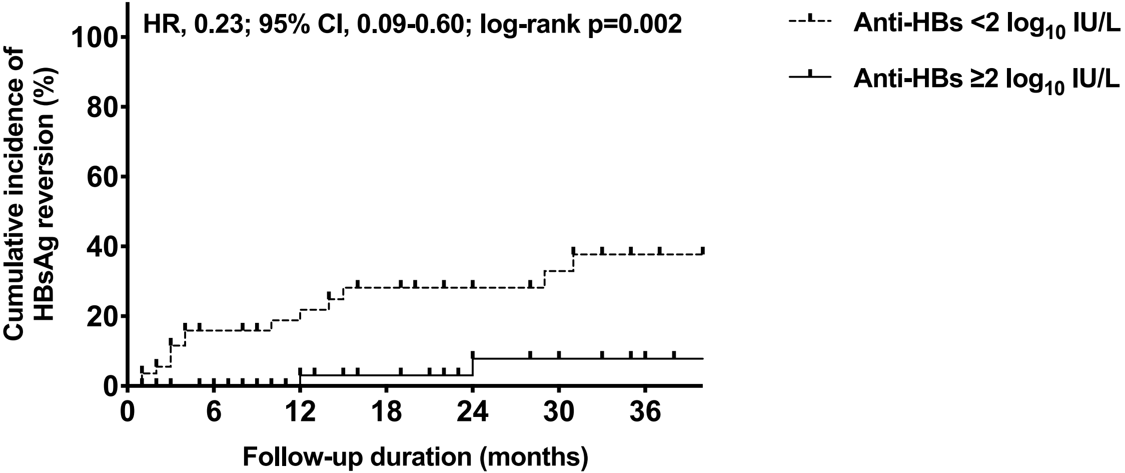

No. at risk

Anti-HBs <2 log_{10} IU/L	55	38	27	22	17	15	10
Anti-HBs ≥2 log_{10} IU/L	57	49	33	27	20	17	14
